# Occurrence of Newcastle Disease and Infectious Bursal Disease Virus Antibodies in Double-Spurred Francolins in Nigeria

**DOI:** 10.1155/2014/106898

**Published:** 2014-11-18

**Authors:** Daniel Oladimeji Oluwayelu, Adebowale Idris Adebiyi, Ibukunoluwa Olaniyan, Phyllis Ezewele, Oluwasanmi Aina

**Affiliations:** ^1^Department of Veterinary Microbiology and Parasitology, University of Ibadan, Ibadan 20005, Nigeria; ^2^Department of Veterinary Anatomy, University of Ibadan, Ibadan 20005, Nigeria

## Abstract

The double-spurred francolin *Francolinus bicalcaratus* has been identified as a good candidate for future domestication due to the universal acceptability of its meat and its adaptability to anthropogenically altered environments. Therefore, in investigating the diseases to which they are susceptible, serum samples from 56 francolins in a major live-bird market (LBM) in Ibadan, southwestern Nigeria, were screened for antibodies against Newcastle disease (ND) and infectious bursal disease (IBD) viruses. Haemagglutination inhibition (HI) test and enzyme-linked immunosorbent assay (ELISA) revealed 25.0% and 35.7% prevalence of ND virus (NDV) antibodies, respectively, while 5.4% and 57.1% prevalence of IBD virus (IBDV) antibodies was detected by agar gel precipitation test (AGPT) and ELISA, respectively. This first report on the occurrence of NDV and IBDV antibodies in apparently healthy, unvaccinated double-spurred francolins from a LBM suggests that they were subclinically infected with either field or vaccine viruses and could thus serve as possible reservoirs of these viruses to domestic poultry. Furthermore, if they are to be domesticated for intensive rearing, a vaccination plan including ND and IBD should be developed and implemented.

## 1. Introduction

Newcastle disease (ND) and infectious bursal disease (IBD) are the two most dreaded viral diseases of poultry in Nigeria as they cause severe economic losses in domestic and wild bird populations resulting from illness, reduced egg production, immunosuppression, and death following infection with pathogenic strains of their respective causative viruses. Despite efforts to prevent and control them over the years, circulation of the causative virus among free-roaming and wild birds has been reported as one of the factors responsible for the sporadic outbreaks of ND and IBD among free-roaming village chickens as well as commercial poultry flocks [1, 2].

Newcastle disease (ND) is an acute, highly contagious, rapidly spreading viral disease affecting birds of all ages [3] and is characterized in chickens by respiratory, circulatory, gastrointestinal, and nervous signs [4]. The clinical signs seen in infected birds vary widely and are dependent on viral factors like pathogenicity (which depends on virulence and tropism of the virus), host factors (species, age, and immune status), concurrent infections, route of exposure, duration and magnitude of the infection dose, and external factors such as social and environmental stress [5]. According to Docherty and Friend [6], it is capable of infecting over 230 species from more than one-half of the 50 orders of birds. These include domestic poultry [7, 8] and wild birds such as house sparrows, hawks, crows, double-breasted cormorants, and waterfowls [9–13]. Wild birds constitute a natural reservoir of low-virulence viruses, while poultry are the main reservoir of virulent strains. Exchange of virus between these reservoirs represents a risk for both bird populations [13]. The most virulent form of ND virus (NDV) causes up to 100 percent mortality in affected flocks [6].

On the other hand, infectious bursal disease (IBD) is a highly contagious immunosuppressive viral infection of chicks (3–6 weeks old) causing severe economic and production losses worldwide [14]. Although turkeys, ducks, guinea fowls, and ostriches may be infected, clinical disease occurs solely in chickens [15]. However, serological evidence of the infection has been reported in free-living wild birds such as cordon bleu and village weaver [16], wild water birds [17], Antarctic penguins [18], cattle egrets [19], and wild turkeys and cranes [20]. Moreover, IBDV antibodies were detectable in the sera of sedentary and migratory wild bird species in Japan, suggesting that they play a key role in the natural history of IBD [21] while the virus was isolated from wild birds in Korea [22].

Although several studies have been conducted on francolins in South Africa [23–25], sparse information exists on the West African-based double-spurred francolin,* Francolinus bicalcaratus* (synonym:* Pternistis bicalcaratus*), which is a gamebird in the pheasant family Phasianidae of the order Galliformes. It is a resident breeder in tropical West Africa and feeds on insects, vegetable matter, and seeds [26]. According to Keith et al. [27], seven Afrotropical francolin species including the double-spurred francolin have been found in Nigeria where they are widely consumed as bush meat. In a comparative biochemical study of meat quality and digestive enzymes, their meat was reported to be tastier, juicier, more palatable, and richer in protein than domestic chicken meat [28]. Based on these desirable qualities of francolin meat, it is needful to consider them as an alternative source of affordable animal protein to the ever-increasing Nigerian human population. Moreover, although they are found mostly in the wild, Mbinkar et al. [29] noted that the species is considered a good candidate for future domestication due to the universal acceptability of its meat and its adaptability to anthropogenically altered environments, which may be occasioned by extensive bush burning and intensive grazing of grasslands. Therefore, since studies on their biology and ecology, which can provide a basis for their eventual domestication, have been conducted [28–31], there is a need to also investigate the infections to which they are susceptible. Apart from few studies which showed that francolins are affected by diseases such as Marek's disease [32], coccidiosis [33], toxoplasmosis [34], and bacterial sinusitis [35], there is sparse information on viral diseases such as ND and IBD in francolins. This study was therefore designed to investigate the presence of NDV and IBDV antibodies in free-living double-spurred francolins caught in the wild and sold at a popular live-bird market (LBM) located in Shasha, Ibadan, southwest Nigeria.

## 2. Materials and Methods

The study was conducted in Ibadan (latitude 7° 23′ N and longitude 3° 56′ E), the capital city of Oyo State, southwest Nigeria. This region is the core of the Nigerian poultry industry with Ibadan being a major city from where poultry (day-old chicks, broilers, and point-of-lay pullets) and poultry inputs (drugs, vaccines, and feed ingredients) are distributed to other parts of the country. The city also has some LBMs, of which the one located at Shasha is popular as it is a sales point for diverse bird species brought by peasant farmers from adjoining rural communities and traders from the northern part of the country that transport cattle, sheep, and goats to southwest Nigeria.

Blood samples collected via the jugular vein between April and August 2012 from 56 double-spurred francolins at Shasha live-bird market were poured into sterile sample bottles without anticoagulant and allowed to clot at room temperature. Separated sera were harvested and stored at −20°C. Although larger sample population of spurred francolins existed in the market and prior consultations were made with the traders on the importance of the project, the number of birds available for bleeding was restricted to 56 in view of the refusal of traders to have their birds bled. The ages of the birds could not be determined.

The 56 sera were screened for antibodies to NDV and IBDV using the haemagglutination inhibition (HI) test and agar gel precipitation test (AGPT) as described by Durojaiye and Adene [36] and Hirai et al. [37], respectively. Positive control ND and IBD sera were obtained from the National Veterinary Research Institute, Vom, Nigeria. Antibodies to NDV and IBDV were also detected and quantified in 42 francolin sera (the remaining 14 sera had been exhausted) using commercial ND and IBD enzyme-linked immunosorbent assay (ELISA) kits (ProFLOK Plus, Synbiotics Corporation, Kansas City, USA), respectively, according to the manufacturer's instructions. Positive and normal control ND and IBD sera were used to validate the tests. As specified by the kit manufacturer, a serum dilution of 1 : 50 was used and optical density (OD) values were read at 405 nm with an ELx800 universal microplate reader (Bio-Tek, Vermont, USA). Valid NDV or IBDV ELISA results were obtained when the average OD value of the normal control serum was less than 0.250 and the corrected positive control value range was between 0.250 and 0.900.

Data obtained were analysed with column statistics using GraphPad prism version 5.0 (GraphPad software, San Diego, CA, USA) and *P* values < 0.05 were considered significant.

## 3. Results and Discussion

Prevalence of NDV antibodies in the tested francolin sera was 25.0% (14/56) and 35.7% (15/42) using the HI and ELISA tests, respectively (Table 1). The HI antibody titers ranged from 1 : 2 to 1 : 32 while mean NDV antibody titer obtained with the ELISA was 3206 (95% CI: 1261–5152). There was a 59.5% agreement between the HI test and the ELISA. Only 3 (5.4%) of the tested sera were positive for IBDV antibodies with the AGPT while 24 (57.1%) were positive using ELISA (Table 2). Mean IBDV antibody titer obtained with the ELISA was 5735 (95% CI: 2919–8550) and there was a 50% agreement between the AGPT and ELISA. Using the ELISA, 28.6% (12/42) of the tested sera had antibodies to both NDV and IBDV.

This study investigated the presence of NDV and IBDV antibodies in free-living double-spurred francolins caught in the wild and sold at Shasha LBM, which is a trading centre where different avian species including indigenous chickens, pigeons, guinea fowls, turkeys, and francolins are sold in Ibadan, Oyo State, southwest Nigeria. To our knowledge, there is no information available on viral diseases of double-spurred francolins which have been suggested as a good candidate for domestication in order to meet the animal protein needs of the Nigerian populace [29]. Therefore, this first report on the detection of NDV- and IBDV-specific IgY antibodies in the sera of free-living francolins in Nigeria is an indication of previous exposure of these birds to the two viruses. Since they were not routinely vaccinated, it is possible that the birds acquired their ND and/or IBD seropositive status through exposure to other infected wild or domestic birds that were shedding the viruses. The seropositivity detected could be due to circulating ND and IBD vaccine viruses which the birds might have contracted through interaction with vaccinated free-range birds or even through operating occasionally around commercial poultry farms. Karesh et al. [38] noted that wild birds in the pet or exotic bird trade have the potential to transmit parasites, bacteria, and viruses which may or may not be pathogenic in their normal host but pose threats when introduced to new geographic locations and new host species. In this study, we observed that wild and domestic bird species were kept by the traders in the same cages and this is consistent with a previous report that ND, for example, is spread by contact between birds and exacerbated by birds being mixed together in rural markets [13]. As the francolins were usually kept at the LBMs for about 3-4 weeks before being sold, it is likely that they acquired the viruses from infected domestic or wild bird species with which they were kept in the same cages.

**Figure 1:**
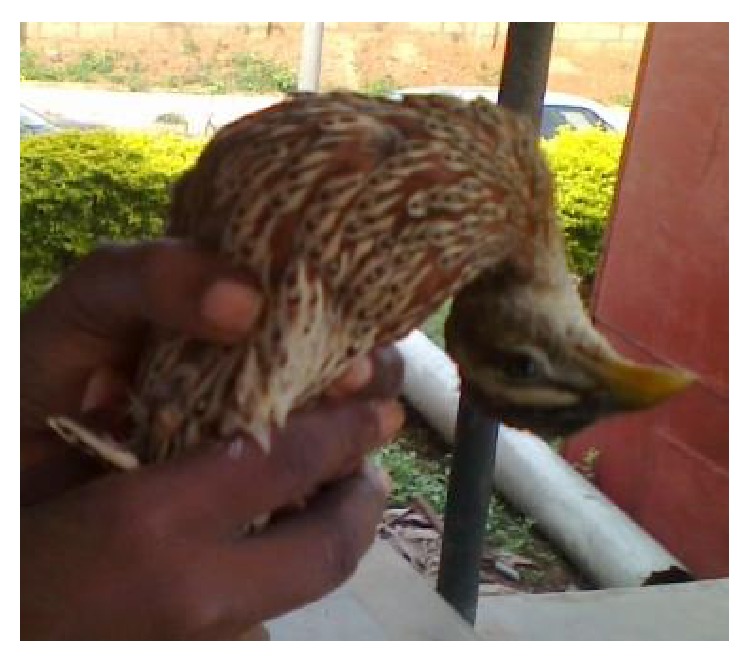
Francolin showing sign of torticollis at the time of blood collection.

Moreover, the francolins tested in this study were all apparently healthy except for one that showed signs of torticollis at the time of sample collection (Figure 1). Therefore, the detection of NDV and IBDV antibodies in their sera suggests that they were subclinically infected and could serve as reservoirs shedding the viruses into the environment. Previous studies have implicated wild birds as possible reservoirs/vectors of these viruses for domestic poultry [16, 21, 39]. It is also possible that the birds possess some host factors responsible for resistance. Moreover, Kim et al. [40] suggested wild-type virus transmission between wild and domestic birds as the origin of the similarity of NDV strains found in wild birds and domestic birds in LBMs.

It is noteworthy that 28.6% (12/42) of the sera tested by the ELISA technique had high titres of both NDV and IBDV antibodies, which is an indication that the birds were coinfected with the two viruses. Additionally, the detection of a higher proportion of positive samples by the ELISA technique compared to the HI test and AGPT shows that it is more sensitive than the latter two tests for detecting NDV- and IBDV-specific antibodies, respectively, in francolin sera. This is consistent with the reports of Bell et al. [41] and Marquardt et al. [42] who also found that the ELISA was more sensitive than the HI test and AGPT for detection of antibodies to NDV and IBDV, respectively.

## 4. Conclusions

This study has shown that free-living double-spurred francolins are susceptible to infection with NDV and IBDV and can serve as reservoirs of these viruses, thus acting as a means of transmission to domestic poultry. Therefore, if they are to be domesticated for intensive rearing as an alternative source of animal protein, a vaccination programme which includes ND and IBD vaccinations should be developed and implemented to protect them from clinical disease. In addition, the detection of NDV and IBDV antibodies in francolins sold at LBMs where they have close interaction with commercial and village chickens and other wild birds warrants continuous surveillance for these diseases because of increased concerns that low-virulence wild bird viruses could become more virulent in domestic bird populations. Further studies to isolate the two viruses from francolins and determine their level of pathogenicity should be conducted.

## Figures and Tables

**Table 1:** Correlation of HI and ELISA for NDV antibodies.

Test	Positive	Negative	Total
HI	10	32	42
ELISA	15	27	42

**Table 2:** Correlation of AGPT and ELISA for IBDV antibodies.

Test	Positive	Negative	Total
AGPT	3	39	42
ELISA	24	18	42
